# Physical breakdown of CH_4_ hydrate under stress: a molecular dynamics simulation study

**DOI:** 10.1186/s13065-024-01191-6

**Published:** 2024-04-27

**Authors:** Xianwu Jing, Li Zhou, Yong Ma, Ziyi Fu, Qian Huang, Zhe Zhang

**Affiliations:** 1 Research Institute of Natural Gas Technology, PetroChina Southwest Oil and Gasfield Company, Chengdu, 610213 Sichuan People’s Republic of China; 2 Shale Gas Evaluation and Exploitation Key Laboratory of Sichuan Province, Sichuan Provincial Department of Science and Technology, Chengdu, 610051 Sichuan People’s Republic of China; 3 Engineering Technology Department, PetroChina Southwest Oil and Gasfield Company, Chengdu, 610081 Sichuan People’s Republic of China; 4 PetroChina Planning and Engineering Institute, Beijing, 100083 China

**Keywords:** CH_4_ hydrate, Stretch, Squeeze, Stress, Order parameters, Deformation

## Abstract

As a solid energy source, CH_4_ hydrate will inevitably break down physically as the result of geological movement or exploitation. Here, the molecular dynamics method was employed to simulate the uniaxial-deformation behavior of structure I (sI type) CH_4_ hydrate under stress. The stress increases regardless of whether the hydrate is stretched or squeezed, and other physical parameters also changed, such as hydrate cage numbers, order parameters, and the number of water molecules. A noticeable difference is observed between the two systems. Upon stretching, the stress immediately recovers to 0 GPa once the hydrate is completely stretched apart. During the squeeze process, the stress is ultimately not zero since solid and liquid are always in contact. When the hydrate is stretched apart, about 5% of water molecules change from solid to liquid, about 7.8% of CH_4_ molecules lose their shelter and become free due to the disintegration of water cages. While in the squeezing process, large cages (5^12^6^2^) are crushed more easily than small cages (5^12^); in the end, about 93.5% of large cages and 73% of small cages are crushed, and approximately 87.5% CH_4_ is released from the cages. In mining CH_4_ hydrates, caution must be exercised, as if the hydrates break as a result of stress, a large release of CH_4_ may pose a security risk.

**Supplementary Information:**

The online version contains supplementary material available at 10.1186/s13065-024-01191-6.

## Introduction

Fossil fuels, such as coal and petroleum, have been widely used across multiple industries for decades and have become indispensable in people's daily lives [1, 2]. As modern society develops rapidly, the demand for energy rises, and the world's energy shortage likewise becomes more acute [3, 4]. Although researchers have endeavored to develop new energy sources such as hydrogen energy [5, 6], hydropower [7], solar energy [8–10], and nuclear energy [11], traditional fossil fuels will continue to play an indispensable role in our energy structure in the foreseeable future, and developing countries in particular rely heavily on these fuels to power economic growth [12]. People may be forced to endure cold temperatures, hunger, disease, poverty, or even war if this fossil energy shortage cannot be solved [13–17]. For humanity's prosperity, it is critical to find a solution to the energy shortage, and to tap into new fossil fuels [18–20].

A new type of clean and efficient energy has been discovered, the methane (CH_4_) hydrate. This is an ice-like inclusion compound formed by water and CH_4_ molecules under high pressure and low temperature [21]. In nature, structure I (sI-type) CH_4_ hydrates are most commonly found in places such as the continental margin, continental slope, and permafrost zone, making CH_4_ hydrate a potent source of energy [22]. At present, large quantities of CH_4_ hydrate have been found in the Shenhu area of the South China Sea [23], the Gulf of Mexico [24], and the Nankai Trough in Japan [25]. According to surveys [26–28], the total carbon reserve of CH_4_ hydrate is more than twice the carbon reserve of conventional fossil energy [29–31], and it has been identified as one of the most promising fossil fuels.

The actual environments in which CH_4_ hydrates are located are characterized by greater complexity than previously believed. For example, once CH_4_ hydrate is formed in the continental margin, it may not be fixed in the same location where it was formed. As a result of external force action such as earthquakes, typhoons, submarine volcanic activity, etc., hydrates may deform due to the stress, additionally, mechanical damage is also inevitable during the CH_4_ hydrate exploration and production process [32, 33]. Furthermore, the massive hydrate dissociation would decrease the strength and modulus of sediment and further resulting in serious hydrate reservoir deformation [34, 35]. If this situation occurs during drilling operations, the stability and bearing capability of hydrate-bearing sediments will greatly decrease, while the effective stress subjected to the mining shaft could significantly increase, resulting in the instability of the hydrate reservoir and well-bore damage [36, 37].

Deformation of hydrates has been studied by many scholars. In Luo’s work [38], they reported the results of shearing with and without depressurization tests on the remolded hydrate-bearing marine sediments from the South China Sea. The results indicate that obvious deformation and strength attenuation should be considered when the depressurization method is used for large-scale hydrate production in silty reservoirs. The deformation characteristics of silty reservoirs induced by hydrate dissociation would be reduced by the high-pressure drainage consolidation, while a higher overburden stress and hydrate saturation would cause more serious deformation. Experimental research is difficult to analyze the mechanism at the micro level. Fortunately, molecular dynamics (MD) simulations can demonstrate the changes in hydrates from a microscopic perspective. Tian et al. [39] investigated the principles of stress–strain evolution under the condition of stretching and squeezing by MD simulation. In their research, the mechanical characteristics and the microstructure evolution mechanism of sI CH_4_ hydrate under different stress conditions were explored to provide theoretical guidance for the practical exploitation of hydrates. However, they only studied the stress and strain behavior of CH_4_ hydrates of different sizes during the tensile process.

Besides studying the change in stress and strain when an external force is applied on sI CH_4_ hydrate, many other properties should also be investigated since it consists of water cages and H-bonds. For example, the phase change of water molecules under stress, the number of h-bonds and water cages, and many other properties. Inspired by Tian’s work, the changes in CH_4_ hydrate properties during stretching and compression were studied using molecular dynamics methods in this work Our research is significant in that it provides clear guidance for the development of methane hydrates.

## Computational details

**Fig. 1:**
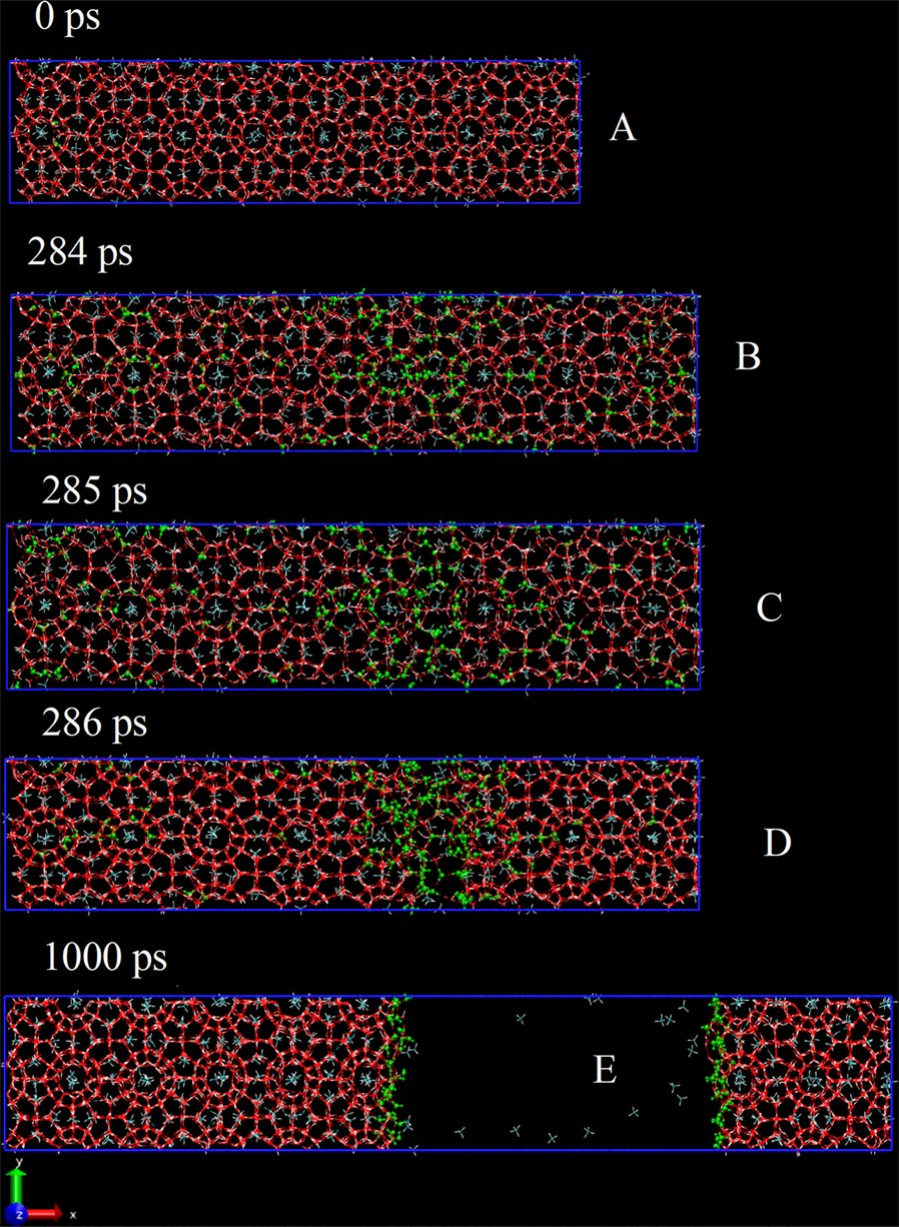
Snapshot of CH_4_ hydrate during the stretching process (the green ball-and-stick model represents liquid water, the rest red one represents solid water)

The unit cell of CH_4_ hydrate was obtained from the literature [40]. Multiwfn [41] was then used to expand it to a supercell as shown in Fig. 1A, the initial box size was 10 × 2.5 × 2.5 nm (X × Y × Z axis). The all-atom TIP4P/Ice water model was used to describe the water [42]; the all-atom CH_4_ model was described by the general AMBER force field (GAFF) [43], and the CH_4_ charge was the RESP charge [44] which was calculated using Multiwfn. MD simulation was carried out using Gromacs software version 2019.6 [45, 46]. To simulate the storage environment as closely as possible, the temperature was set at 270 K. Furthermore, there was an anisotropic zero-pressure isobaric-isothermal NPT ensemble, in which, all sides of the orthogonal cell were allowed to vary independently. To ensure that the simulation boxes remain rectangular, the pressure was set at 10 MPa in the XX, YY, and ZZ directions, and 0 MPa in other directions. The corresponding equations of motion are based on a Parrinello-Rahman barostat [47] and a V-rescale thermostat [48]. In the present simulations, periodic boundary conditions were applied and in the X direction, the deformation velocity was 0.005 nm/ps and − 0.005 nm/ps to simulate the processes of stretching and squeezing. The total simulation time was 1000 ps for both the two simulations.

## Results and discussion

### Stretch and squeeze deformation simulation.

As CH_4_ hydrate is solid, we assume that similar to that of stretch or squeeze biscuits, the solid CH_4_ hydrate will become elongated or squashed and eventually rupture in a short period of time when being stretched or squeezed. Here, we took screenshots at different time points during the simulation, and further, the CHILL + algorithm [49] was used to identify the quantity of water molecules in different states, including hydrate, hexagonal ice, cubic ice, liquid water, and between solid (both hydrate and ice) and liquid.

Firstly, uniaxial stretch deformation is simulated, and screenshots at different time points are shown in Fig. 1, and Additional file 1: Video S1 illustrates this process vividly. With the stretching simulation, the X-axis is extended smoothly; with the simulation time elapses to 284 ps, as shown in Fig. 1B, the perfect CH_4_ hydrate shows inconspicuous cracks, some liquid water molecules can be seen; then, obvious cracks appear at 285 ps, as shown in Fig. 1C; and a clear fracture surface can be seen at 286 ps, as shown in Fig. 1D, liquid water molecules are mainly distributed at fracture surfaces, suggesting the perfect hydrate was stretched apart in an instant. The distance between the fracture surfaces increases as the simulation time increases. However, the other parts remain unchanged, even when the simulation time elapses to 1000 ps, as shown in Fig. 1E. The vacuum zone is extended, but the overall other states are not significantly different from those at 286 ps, liquid water is only distributed at the fracture surface, while the other water molecules are in the solid sI hydrate form. This indicates that the fracture surface is the only place where the phase change occurs..

**Fig. 2:**
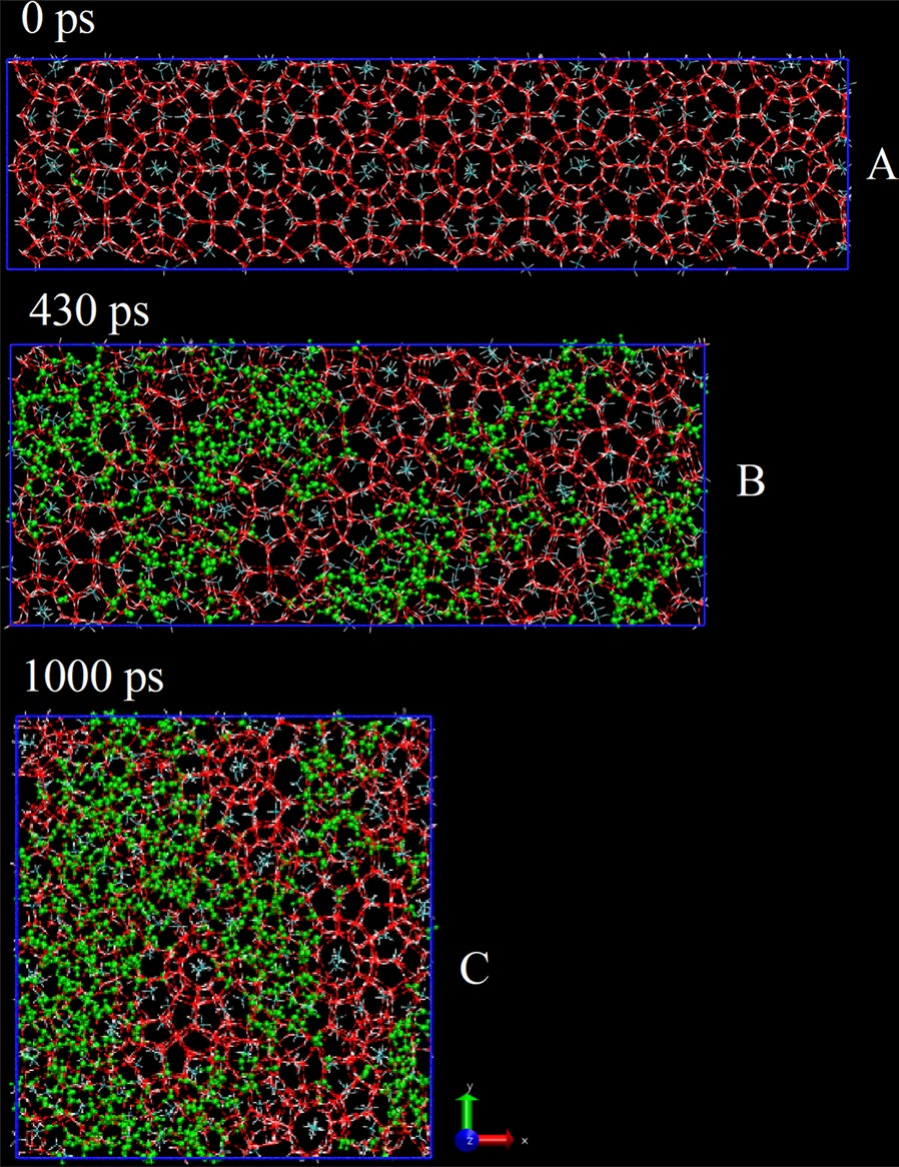
Snapshot of CH_4_ hydrate in the squeezing process (the green ball-and-stick model represents liquid water, the rest red one represents solid water)

To further investigate the deformation of solid CH_4_ hydrate under external force, we modified the force direction and examined the change of CH_4_ hydrate under external pressure. It can be seen from Fig. 2 that as the simulation begins, the box is squeezed and the X-axis is shortened. At around 430 ps, as shown in Fig. 2B, an obvious crack forms, and some cages begin to break, the arrangement of water molecules loses its polygonal structure. Though some cages can still be seen and CH_4_ molecules are bound in cages, a large amount of liquid water can also be seen. Upon reaching 1000 ps, as shown in Fig. 2C, a small number of cage structures with regular water molecule arrangement and a large number of water molecules with irregular distribution can both be seen, indicating that significant physical change have taken place. This change can be seen in Additional file 2: Video S2 vividly.

**Fig. 3:**
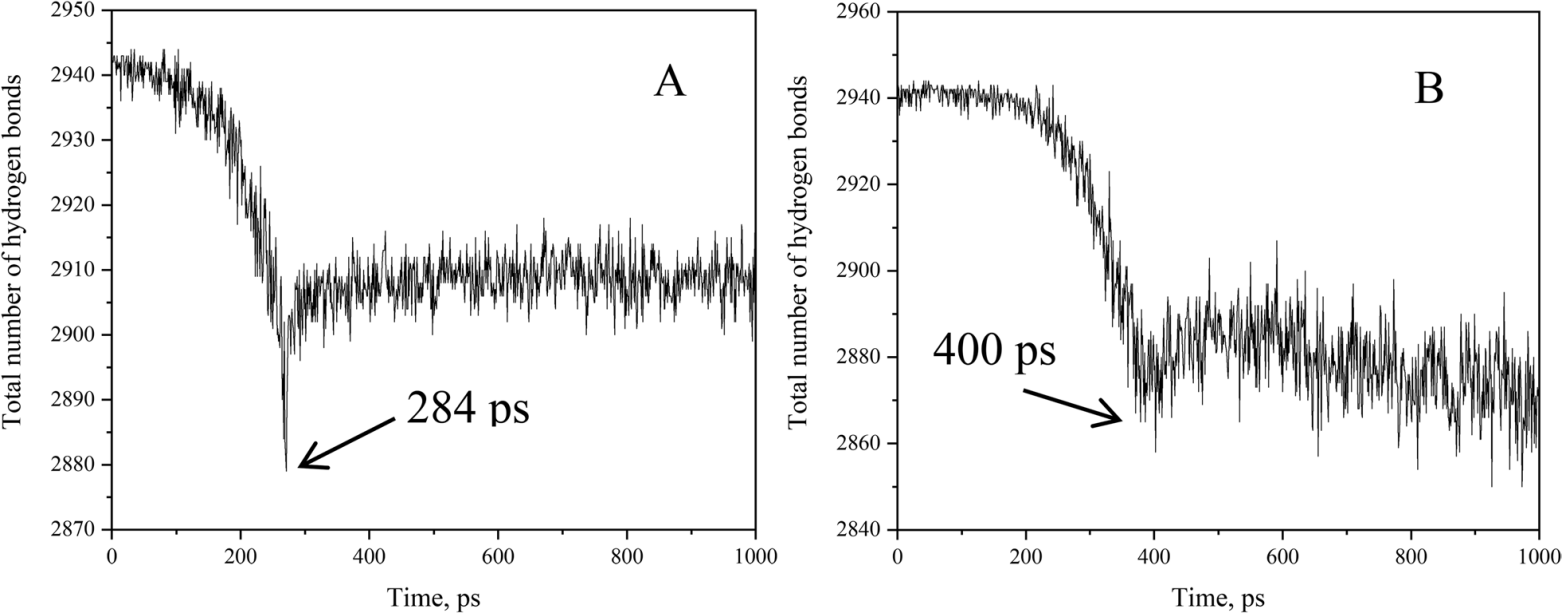
Changes in the number of H-bonds during stretching and squeezing

Considering the above results, we are interested in the micromolecular changes that occur. In previous report, in the bulk phases of ice or hydrates, each water molecule can form four H-bonds with surrounding water molecules [50, 51].here, can a water molecules at the fracture surface or in the bulk phase still form four H-bonds when the hydrate is stretched apart or crushed? A discussion is provided in Fig. 3.

According to Fig. 3A, as the stretch simulation starts, the total number of H-bonds declines, and when simulation time reachess 284 ps, the solid hydrate is stretched apart instantaneously, which leaves the number of H-bonds can be partially restored due to the elastic changes of the solid substance, but it can never be restored to the original value. The water molecules at the fracture surface no longer maintain contact with one another, and of course, a water molecule can't form the full four H-bonds. In contrast, the number of H-bonds gradually decreases once the squeeze simulation begins, indicating that the cage structure of the hydrate is being disrupted. When the simulation reached 400 ps, the solid hydrate was crushed and liquid appeared inside the box, shown in Fig. 3B. Since the water molecules remain in contact during the squeezing process, the hydrate phase state changes, and not all water molecule can form four H-bonds with surrounding water molecules. When the solid hydrate is totally crushed, the number of H-bonds remains basically unchanged. This also proves that liquid water molecules do not always form four H-bonds with surrounding water molecules.

### Quantity of water molecules in different states

**Fig. 4:**
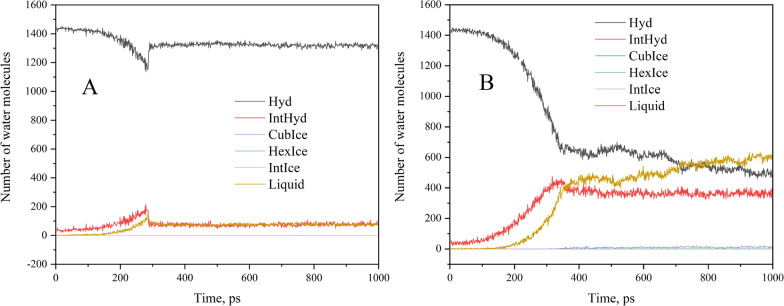
Number of water molecules belonging to different phase states during stretching (**A**) and squeezing (**B**)

According to the research above, some cages formed by water molecules can remain in the initial solid state when stretched or squeezed, however, some cages are destroyed, and the water molecules originally belonging to the solid state change to the liquid state. Thus, how many water molecules in different phases would be? The CHILL + algorithm was also used to determine the number of water molecules in the following states: hydrate, hexagonal ice, cubic ice, liquid water, and between solid (both hydrate and ice) and liquid. As can be seen from Fig. 4, there are no cubic ice or hexagonal ice the only water molecules present are sI hydrate and liquid water molecules in the stretching and squeezing process.

As shown in Fig. 4A, when stretching begins, the number of solid hydrate water molecules decreases while the number of liquid state water molecules increases gradually, the number of water molecules between the hydrate and liquid surface also increases. Due to elastic deformation, the deformed caged hydrate partially recovered when the moment CH_4_ hydrate is stretched apart, the number of solid hydrate water molecules increased and the number of liquid hydrate water molecules decreased. When the hydrate is stretched apart, the system will not change significantly over time.

In the same way, during the squeezing process, there are only solid and liquid state water molecules are present. As shown in Fig. 4B, once squeeze begins, the number of hydrate water molecules decrease significantly, while the liquid water molecules number increases. Naturally, the number of water molecules at the hydrate-liquid interface also increases gradually. When the simulation time reaches 487 ps, most of the solid hydrate cages are crushed and this part of the water molecules that used to belong to the solid hydrate become liquid. Increasing the simulation time does not seem to affect the system in any noticeable way.

### Box sides length and volume change

**Fig. 5:**
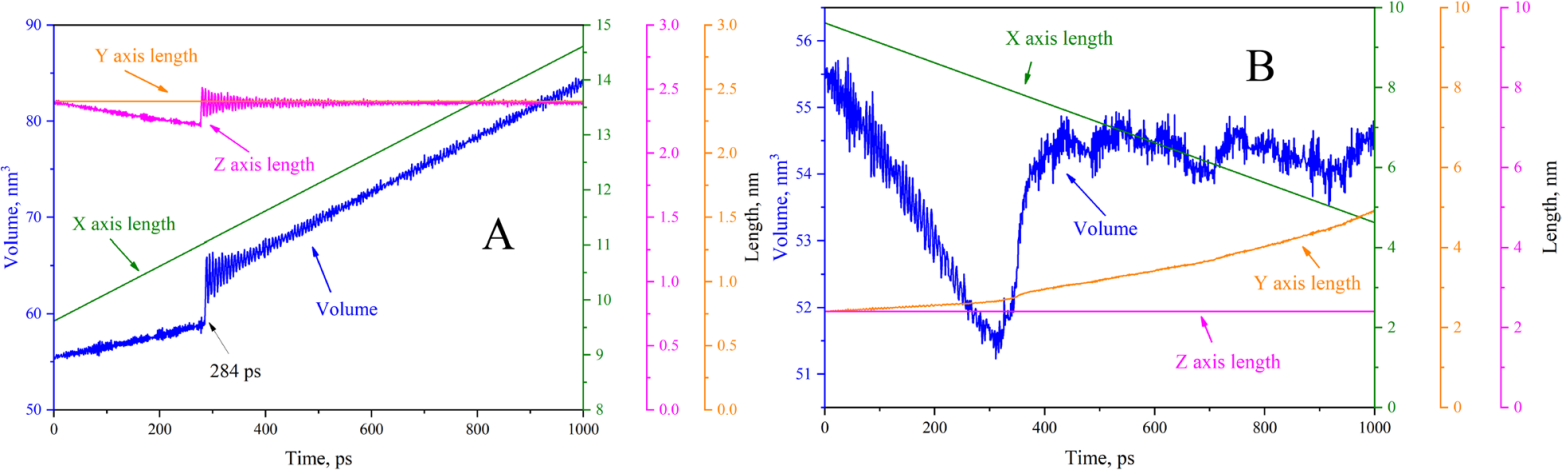
The length of box sides and volume changes during the stretching (**A**) and squeezing (**B**) process

As shown in Figs. 1, 2, and the videos in the supporting information, the boxes change significantly when stretched and squeezed. The X, Y, and Z axis lengths, as well as the box volumes, all warrant further study, as shown in Fig. 5..

As the stretch simulation begins (Fig. 5A), the volume of the simulation box increases gradually; the X-axis is prolonged uniformly; the Z-axis becomes slightly deformed but in a uniform manner due to elasticity; and the length of the Y-axis remains constant. That is, two coordinate axes deform in the stretching process, thus resulting in changes to the volume. The stretching process produces an interesting phenomenon that when the simulation time reaches 284 ps, the solid CH_4_ hydrate is stretched apart instantly, the volume increases suddenly and the Y-axis length returns to the initial value with slight changes due to elasticity. The sudden change in volume and the length of the Y-axis are synchronous, that is, indicating that most elastic deformation occurs along the Y- axis. It can also be concluded from the volume at the moment in which a fracture occurs that the maximum volume deformation amplitude of CH_4_ hydrate under tensile is about 7%. Once the deformation amplitude exceeds 7%, CH_4_ hydrate will be stretched apart.

However, just as if one were to squeeze a biscuit, during the squeezing process of CH_4_ hydrate, the volume is reduced in a smooth manner, whereas the X-axis is shortened at a constant speed, as shown in Fig. 5B. Meanwhile, the Y axis is extended slightly, and the length of the Z axis remains constant. Therefore, the volume change during squeezing process is only related to the X-axis and Z-axis. As the simulation time reaches around 430 ps, the CH_4_ hydrate is slowly crushed, and its volume increases due to the recovery of elastic deformation, as shown in Additional file 2: Video S2. However, in terms of amplitude of change of the volume, when CH_4_ hydrate is compressed to 93.5% of the original volume, it will be crushed.

### Tensile and squeezing stress

**Fig. 6:**
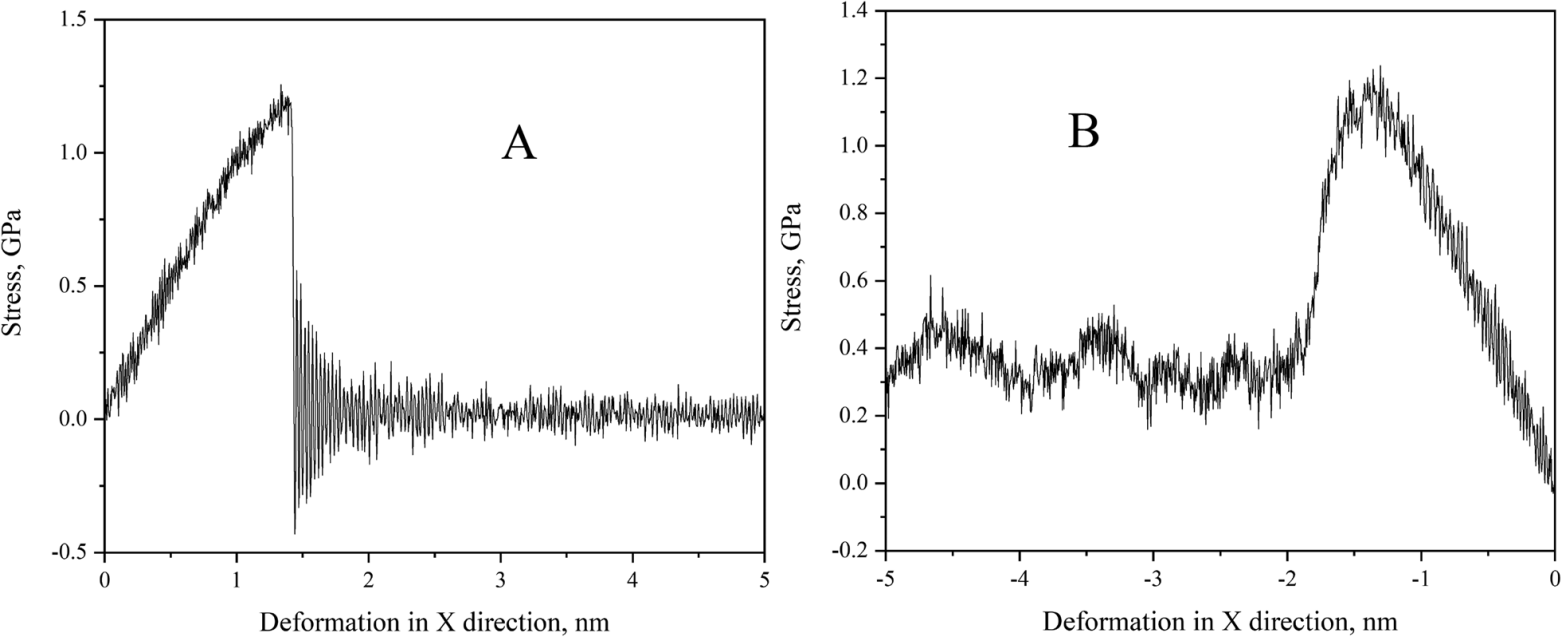
Stress change during stretching (**A**) and squeezing (**B**) process

To discuss the change in stress during stretching and squeezing, we will look at the length change in the X axis as it is stretched or shortened at a constant speed as abscissa, to discuss the change in stress during the stretching and squeezing processes. The length-stress relationship for stretching and squeezeing is presented in Fig. 6.

When stretching begins, shown in Fig. 6A, the stress rises sharply. At a stretch length of 1.42 nm, the stress reaches around 1.2 GPa. As the CH_4_ hydrate continues to be stretched, it fractures and the stress decreases to 0 GPa in as instant. Due to the elasticity of the solid CH_4_ hydrate, the stress fluctuates, resulting in a serrated stress curve. This indicates a reciprocating oscillation direction and is in line perfectly with the information presented in Additional file 1: Video S1. However, upon changing the stress direction of deformation, it can be seen that the stress change shown in Fig. 6B is significantly different from Fig. 6A. During the squeezing process, the length of the X-axis decreases while the stress increases continuously. Around 480 ps in the simulation, the stress reaches the maximum value, which is about 1.2 GPa. As the simulation continues, the CH_4_ hydrate is gradually crushed, and the stress gradually decreases. During this process, there is no sudden change in stress and the stress did not return to 0 GPa, but remained around 0.3 GPa in the final state.

Figure 6A shows no contact between the two fault blocks after the solid CH_4_ hydrate is stretched apart, so the stress will be 0 GPa. However, in Fig. 6B, even though the CH_4_ hydrate is slowly crushed, resistance remains between water molecules in the squeezing process, so the stress recovery is not instantaneous. The videos also demonstrate that these phenomena are in line with scientific theory and daily observations.

### Cage numbers

As we know, sI CH_4_ hydrate is a type of solid substance, with a good deal of cage-shaped structure formed by water molecules in a certain order through the help of H-bonds, and CH_4_ is bound in all cages [40]. The hydrates undergo physical changes as they are separated or crushed, the water and CH_4_ molecules themselves do not undergo any chemical changes, but rather the arrangement of the water molecules changes. Here, we use the hierarchical topology ring (HTR) algorithm [52] to recognize cage structures with high efficiency and accuracy.

**Fig. 7:**
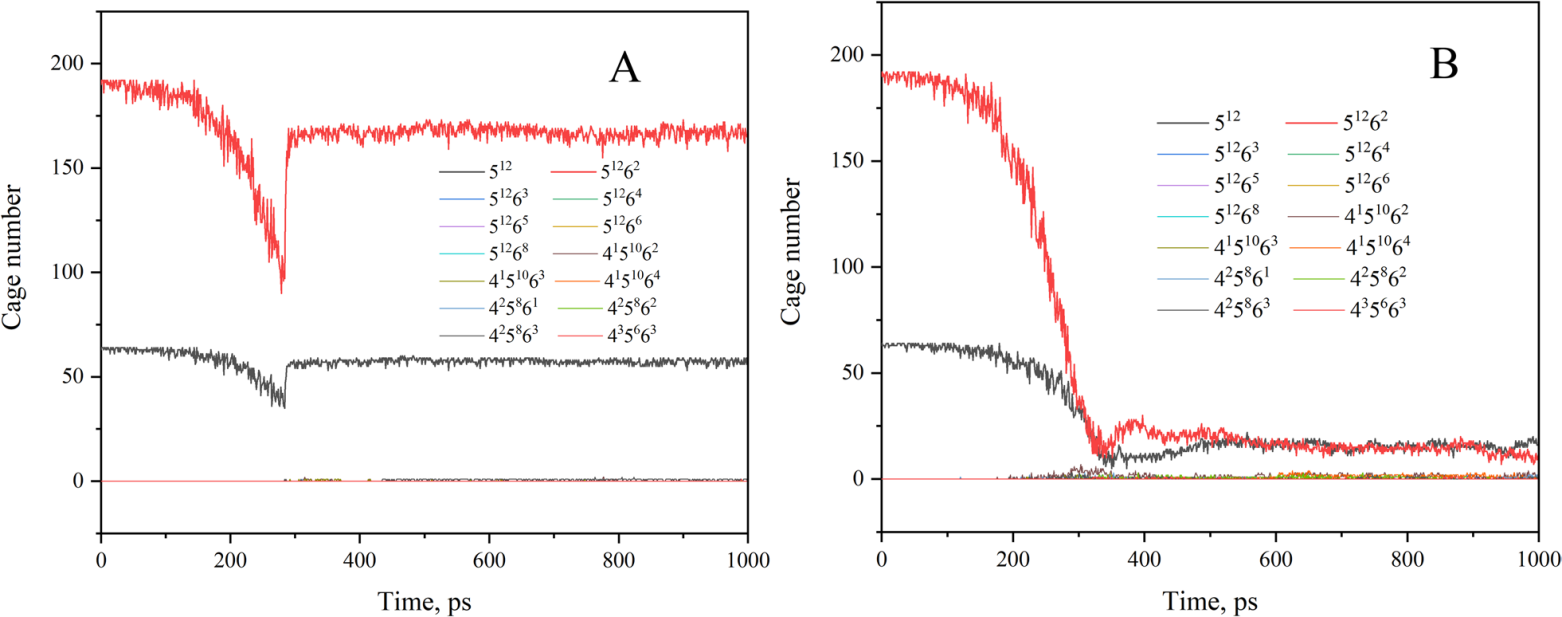
Cage number changes during the stretching (**A**) and squeezing (**B**) process

According to Fig. 7, except for the 5^12^ and 5^12^6^2^ cages, the number of other types of cages, such as 5^12^6^3^ and 4^2^5^8^6^3^ and others, are almost zero, and the number of 5^12^6^2^ cages is around three times that of the 5^12^ cages. This shows that the hydrate is a perfect sI type hydrate [40], regardless of whether it is stretched or squeezed. This being the case, the following study did not consider the minority cages.

As can be seen in Fig. 7A, the number of cages decreased gradually over the simulation course, indicating that the cages formed by water molecules become deformed, the arrangement of water molecules does not comply with standard of the 5^12^ or 5^12^6^2^ cages. When the simulation time reaches 285 ps, the hydrate is completely stretched apart instantaneously, and some cages rebound due to the elastic recovery. However, the cage-like structure of the broken solid portion keeps the number of cages from oscillating significantly, regardless of whether the solid portion is deformed by elastic forces slightly. It is clear from Fig. 7A that approximately 7.8% of the cages are destroyed, and therefore the CH_4_ molecules originally bound in the cages lose their hiding place, that is, 7.8% of the CH_4_ molecules originally bound in the hydrate are released.

Similarly, in Fig. 7B, as the simulation begins, the number of cages start to decrease, indicating that the hydrate cage is crushed during the squeezing process. It is evident that the cage structure is not that of the sI types of hydrate, When the simulation time reaches approximately 387 ps, the number of cages declines to a very low level. Even if the simulation time were extended, the number of cages would not change significantly. In conclusion, a large number of cages will disintegrate during the squeezing process, and only a very small number of water molecules will remain in solid sI type hydrates. According to Fig. 7B, approximately 87.5% of gas molecules will be released due to the change in cage number.

In both stretching and squeezing processes, the number of cages decreases significantly, what phase will the non-solid water molecules be in? Furthermore, how many water molecules remain solid and how many are transformed to other phases?

### Order parameters

From the above results, it can be concluded that as soon as stretching or squeezing starts, hydrates begin to deform and water molecules undergo phase changes, that is, the arrangement of water molecules has changed. The micro configuration of the water system can be discussed by using order parameters. The tetrahedral order parameter (F3) and four-body order parameter (F4) are both commonly used to analyze the arrangement of H_2_O molecules [53].

F3 is defined by Eq. 1:

1 $$F_3 = \frac{1}{n_i (n_i - 1)/2}\sum_{j = 1}^{n_i { - }1} {\sum_{k = j + 1}^{n_i } {(\cos \theta_{jik} |\cos \theta_{jik} | + \cos^2 (109.47))^2 } }$$

where $\theta_{jik}$ is the angle constituted by three adjacent water oxygen atoms ($j$,$i$,$k$), and the $i$ th water oxygen atom is in the center. The value of 109.47° corresponds to the angle between the central vertex line of a regular tetrahedron and represents the maximum value of the minimum angle among four vectors in three-dimensional space.

As a classical order parameter, F4 is defined as Eq. 2:

2 $$F4 = \frac{1}{n_i }\sum_{i = 1}^n {\cos 3\varphi_i }$$

where $\varphi_i$ represents the dihedral angle between the oxygen atoms of two adjacent molecules and the outermost hydrogen atoms and $n$ specifies the number of oxygen atom pairs within 0.35 nm of H_2_O molecules.

The average value of F3 is 0.1 for liquid H_2_O and approximately 0.01 for solid H_2_O (including ice and sI CH_4_/CO_2_ hydrate); F4 is approximately − 0.04, − 0.4, and 0.7 for liquid water, sI ice, and sI CH_4_/CO_2_ hydrate, respectively [53].

**Fig. 8:**
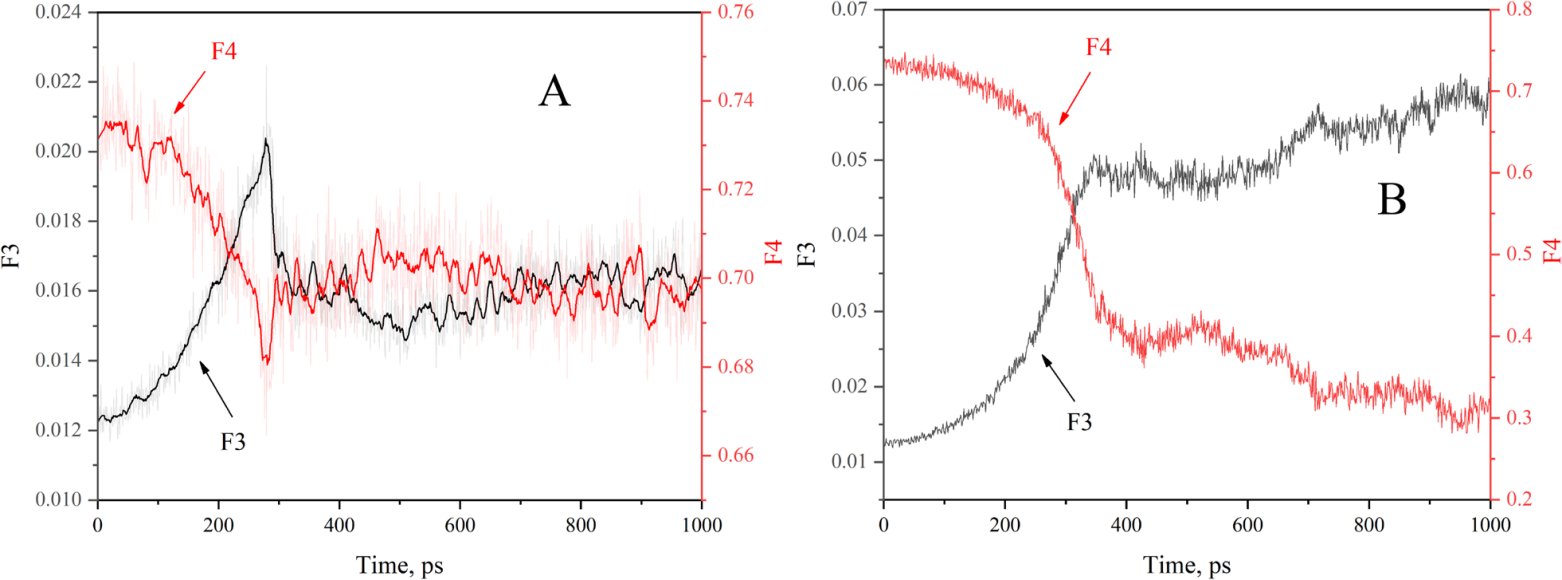
Order parameters vs. simulation time during stretching (**A**) and squeezing (**B**) process

In Fig. 8A, the initial F3 order parameter is approximately 0.012 and the F4 order parameter is approximately 0.735, indicating that the hydrate is a perfect sI type one. Upon stretching, the F3 order parameter demonstrates an upward trend, whereas the F4 order parameter shows a downward trend, suggesting the deterioration of crystal structure. However, the amplitude changes in F3 and F4 are quite small, indicating that only a very small number of water molecules underwent phase changes. As long as the CH_4_ hydrate is stretched apart, the F3 and F4 order parameters remain basically unchanged.

Figure 8B shows that the F3 order parameter increases gradually that during the squeezing process, while the F4 order parameter decreases gradually. Upon reaching 487 ps, there is an inflection point in the change trend of the order parameter As the simulation continues, the F3 order parameter rises slowly, and the F4 order parameter decreases slowly, showing solid water molecules are constantly changing into liquid water during squeezing. The changes in order parameters also confirm that water molecules undergo state changes during the stretching and squeezing processes.

## Conclusions

Stretching or squeezing the perfect sI CH4 hydrate causes it to undergo physical deformation, some cages formed by water molecules rupture, and partial solid water molecules change into liquid. When the CH_4_ hydrate is stretched apart, only the water molecules at the fracture surface are liquid and a large number of cages are crushed and a large number of solid phase water molecules become liquid during the squeezing process In either case, the CH_4_ originally bound in the cages will be released due to the damage of the cage structure. Squeezing, however, releases a greater amount of gas molecules than stretching.

It can be reasonably inferred that it is possible for flammable CH_4_ to leak into the atmosphere during the development of CH4 hydrate, which could pose safety risks. Thus, sufficient precautions must be taken during the process of extraction of CH_4_ hydrate to avoid accidents in which a large amount of CH4 hydrate is exposed to external forces and overflows.

### Supplementary Information

**Additional file 1:** Trajectory video in the stretching process.

**Additional file 2:**  Trajectory video in the squeezing process.

## Data Availability

All the data that support the findings of this study are available on request from the corresponding authors.
